# Recording animal vocalizations from a UAV: bat echolocation during roost re-entry

**DOI:** 10.1038/s41598-018-26122-z

**Published:** 2018-05-17

**Authors:** Laura N. Kloepper, Morgan Kinniry

**Affiliations:** Department of Biology, Saint Mary’s College, Notre Dame, IN USA

## Abstract

Unmanned aerial vehicles (UAVs) are rising in popularity for wildlife monitoring, but direct recordings of animal vocalizations have not yet been accomplished, likely due to the noise generated by the UAV. Echolocating bats, especially *Tadarida brasiliensis*, are good candidates for UAV recording due to their high-speed, high-altitude flight. Here, we use a UAV to record the signals of bats during morning roost re-entry. We designed a UAV to block the noise of the propellers from the receiving microphone, and report on the characteristics of bioacoustic recordings from a UAV. We report the first published characteristics of echolocation signals from bats during group flight and cave re-entry. We found changes in inter-individual time-frequency shape, suggesting that bats may use differences in call design when sensing in complex groups. Furthermore, our first documented successful recordings of animals in their natural habitat demonstrate that UAVs can be important tools for bioacoustic monitoring, and we discuss the ethical considerations for such monitoring.

## Introduction

As UAV technology has become more accessible, many biologists are turning to these airborne platforms for wildlife recording^1^ to conduct surveys of birds^2–5^, reptiles^6^, fish^7^, and mammals^8–11^. Although recently a UAV recorded avian song projected through a speaker^12^, animal vocalizations in their natural habitats have not yet been recorded by UAVs, likely due to their noise profile^13,14^. Echolocating bats, particularly the Brazilian free-tailed bat (*Tadarida brasiliensis*), are excellent animals for UAV recording. These bats produce ultrasonic, FM signals while flying at high speeds^15,16^ and high altitudes^17^, and utilize a flexible echolocation system that adapts to flight behavior and target range^18–20^. Brazilian free-tailed bats are social animals, forming large colonies numbering in the millions^21^, and demonstrate complex group dynamics^22^, including rapid, irregular flight at estimated speeds up to 128 km/h during morning roost re-entry as bats descend from the sky^15^. At these speeds, bats should experience echoes Doppler shifted by several kilohertz, resulting in ranging errors^23,24^, which could prove problematic for a bat flying toward the ground. Additionally, bats face the challenge of avoiding collisions with nearby conspecifics, so bats re-entering the roost face competing sensory challenges. The roost re-entry behavior of *Tadarida brasiliensis* presents a unique opportunity to explore what, if any, call modulations are made during this rapid, close entry to the roost when group cohesion is seemingly lacking.

The purpose of this study was to use UAV technology to conduct acoustic recordings from bats during re-entry to examine if call parameters change according to flight altitude. We equipped a UAV with an ultrasonic microphone and custom baffle, and collected acoustic signals from bats at five flight altitudes. We report on two key findings: animal vocalizations recorded from a UAV, and the first published description of bat echolocation parameters during cave re-entry when bats are flying in groups.

**Figure 1:**
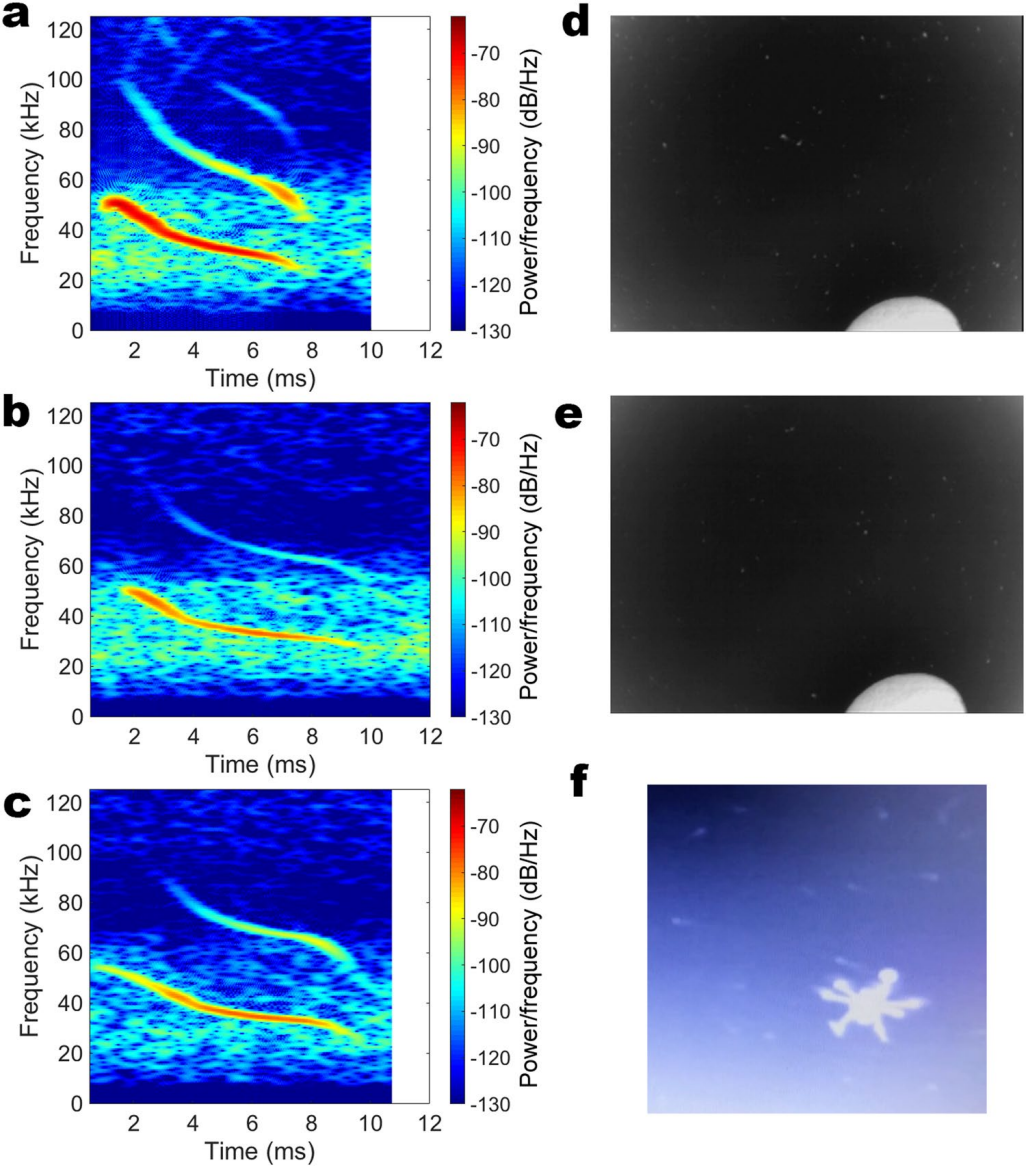
Example spectrograms and thermal imagery of echolocation calls recorded by the UAV. A–C: Spectrograms of sample bat calls recorded at different UAV flight heights and with different SNRs. (**a**) 30 m flight height, 15.19 SNR, (**b**) 40 m flight height, 6.96 SNR, (**c**) 5 m flight height, 3.18 SNR. All calls were filtered using a 16 kHz, 8th order high-pass Butterworth filter. Spectrogram settings: window, 256; overlap, 255; sampling points, 512. D–F: Sample frames from thermal imagery video depict that bats move away from UAV during flight, but still remain within range for detection and flight tracking. (**d**) UAV powered on, but stationary on ground (bats are small white dots), (**e**) UAV hovering at 5 m altitude (bats are small white dots), (**f**) ground-based image of bats with UAV hovering at 10 m altitude. The component of the UAV producing a thermal signature near the rotors is approximately 10 cm in diameter, and *Tadarida brasiliensis* have an average wingspan of 30 cm^23^, so bats approximately 1/3 the size of the rotor are close in altitude to the UAV.

**Figure 2:**
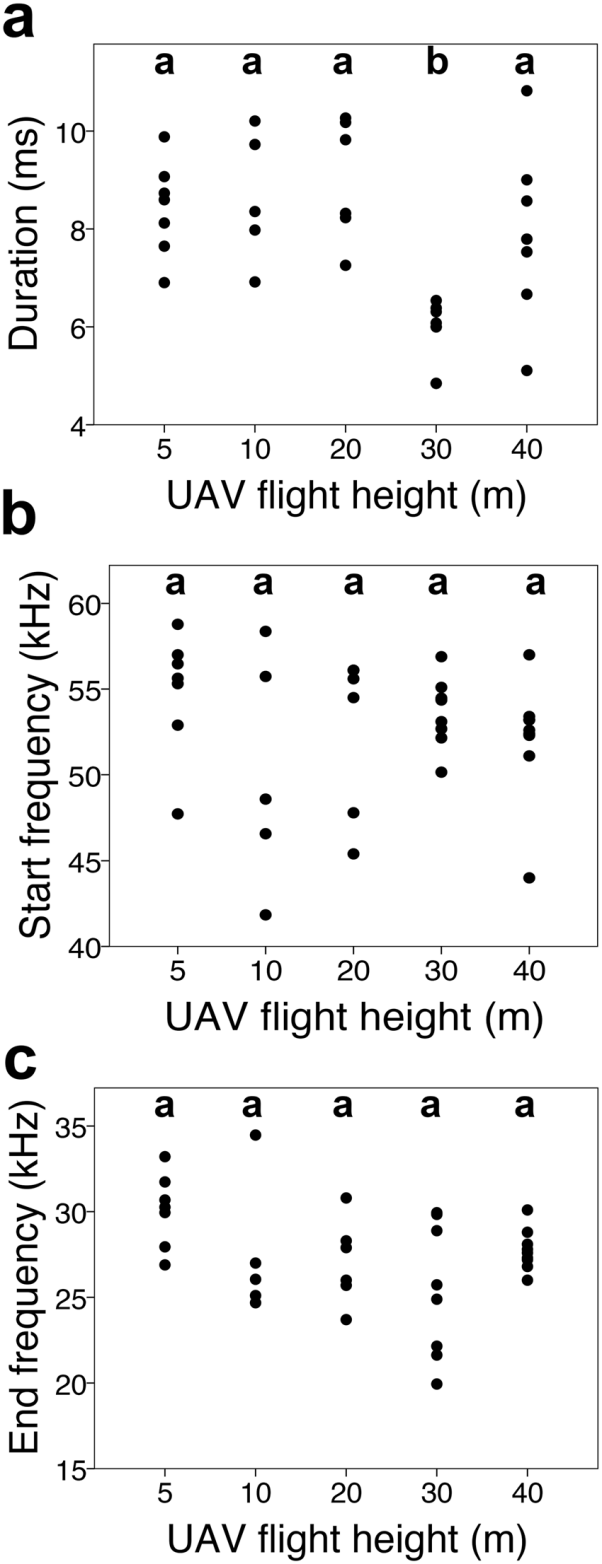
Scatterplots of (**a**) duration (ms), (**b**) start frequency (kHz), and (**c**) end frequency (kHz) for echolocation signals recorded at each UAV recording height (m). Values were determined from the −20 dB values relative to the peak for each signal. Groups sharing a letter indicate the mean ranks via Mann-Whitney U tests were not significant (p > 0.05).

**Figure 3:**
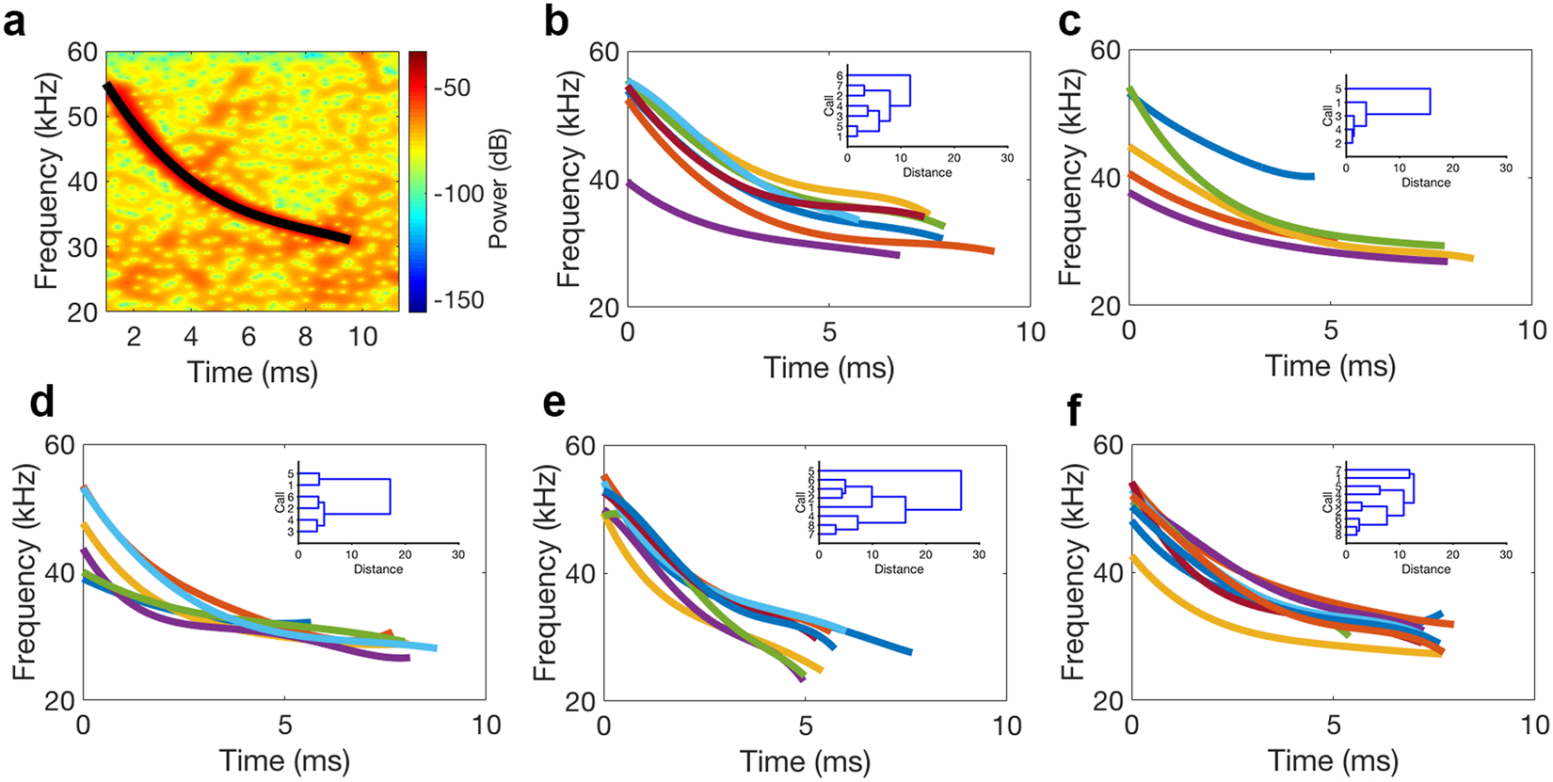
Traces of the time-frequency curvature shape for calls from each altitude. (**a**) Example call spectrogram with the time-frequency trace (black solid line) extracted. (**b**–**f**) time-frequency traces and agglomerative hierarchical clustering results of trace shape (inset) for (**b**) 5 m, (**c**), 10 m, (**d**), 20 m, (**e**), 30 m, and (**f**), 40 m. Cophenetic correlation coefficients for the clustering are: 5 m, 0.735; 10 m, 0.953; 20 m, 0.929; 30 m, 0.733; 40 m, 0.753.

From our total of 84 minutes recording with the UAV, 3,847 echolocation calls were extracted during the re-entry of *Tadarida brasiliensis*, with SNRs ranging between −0.88 and 15.19 dB. All calls detected were FM downsweeps (see Fig. 1 for representative spectrograms). After filtering to ensure we analyzed high quality calls (defined as signal to noise ratios greater than 3 dB) from different bats, we obtained a total of 35 calls for final analysis across five UAV flight altitudes (Table 1). Levene’s test showed that the variance for pulse duration was equal across all heights [duration: F(4,30) = 0.977, p = 0.44]; but variance in start and end frequency was not equal across heights [start frequency: F(4,30) = 3.086, p = 0.031; end frequency: F(4,30) = 2.930, p = 0.037]. The lowest variance for start and end frequency occurred at the second highest and highest recorded altitude, respectively (Table 1). Call duration differed significantly across recording height (Kruskal-Wallis: H(4) = 18.062, p = 0.001), but start and end frequency did not differ significantly across recording height (Kruskal-Wallis: start frequency, H(4) = 3.796, p = 0.434; end frequency, H(4) = 8.868, p = 0.064). The mean rank of the duration at 30 m was significantly lower than at 5 m (U = 0, p = 0.001), 10 m (U = 0, p = 0.003), 20 m (U = 0, p = 0.002), and 40 m (U = 6, p = 0.004), (Fig. 2). All other comparisons via Mann-Whitney U were not significant (p > 0.05). Agglomerative hierarchical clustering results (Cophenetic correlation coefficients: 5 m, 0.735; 10 m, 0.953; 20 m, 0.929; 30 m, 0.733; 40 m, 0.753) based on the time-frequency trace of each signal demonstrated inter-individual differences in call shape, with the greatest differences between calls, or the greatest call variability, for 30 m recording altitude (Fig. 3).

**Table 1:** Summary statistics for the parameters (duration, start frequency, and end frequency) extracted from the calls in the dataset.

Altitude (m)	N	Duration (ms)	Start Frequency (kHz)	End Freqency (kHz)
5	7	8.42 ± 0.97	54.83 ± 3.60	30.10 ± 2.15
10	5	8.63 ± 1.33	50.22 ± 6.76	27.46 ± 4.02
20	6	9.01 ± 1.25	52.58 ± 4.73	27.07 ± 2.47
30	8	5.88 ± 0.66	53.61 ± 2.05	25.38 ± 3.92
40	9	7.87 ± 1.57	52.13 ± 3.44	27.74 ± 1.19

Values under each parameter indicate mean +/− SD.

Our results demonstrate the first successful recording, via UAV, of vocalizations produced by animals in their natural environment. During our 84 minutes of recordings, we identified 3,847 echolocation calls. All of our calls were steep FM downsweeps, as opposed to the shallow FM search calls this species often makes while flying in the open^18,19,25^, which agrees with prior published information on *Tadarida brasiliensis* call shape close to the ground and during target pursuit^18,20^. Our call parameters were different than those previously reported for this species^20,26^, but since Brazilian free-tailed bats demonstrate flexible echolocation that varies according to flight behavior^18^ and geographic location^25^, these results are not surprising. Because there are no other published call characteristics during high-speed roost re-entry for this species, nor for any other bat species, we have no other data available for direct comparison. We therefore consider our data an initial dataset for acoustic behavior during high-speed roost re-entry while flying in groups, and expect additional datasets of acoustic re-entry to demonstrate further variation in call parameters.

In our recordings, we found changes in call duration (Fig. 2) and call shape (Fig. 3) according to UAV flight height. Call shape had the greatest variability at our second highest recorded altitude, 30 m (Fig. 3). Variance in starting and ending frequencies was also greatest at the higher (30 and 40 m) UAV recording altitudes (Table 1). Inter-individual variation in call characteristics has been documented in other echolocation studies^26,27^, and is further enhanced during group flight, when bats can shift frequencies away from conspecifics to avoid acoustic jamming^19,28^. During *Tadarida brasiliensis* re-entry behavior, bats fly in dense groups, so a strategy of creating a signal different from conspecifics would be advantageous for detecting echoes in a cluttered acoustic environment–an environment cluttered by nearby bats and the close proximity of the ground. Additionally, because these bats demonstrate flexible echolocation, it is likely individuals make altitude-specific adjustments in call design during re-entry to compensate for Doppler-shifted echoes. Further studies investigating roost approach behavior for a single individual could elucidate if there are any universal adjustments in call parameters that vary during re-entry approach.

Because our study represents the first description of using a UAV to collect acoustic recordings from animals in their natural habitat, it is important to emphasize ethical considerations. Although UAVs are considerably safer and less noisy than manned aircraft^13^, they are not without their impacts to wildlife and humans. Animals typically show a behavioral response to UAVs, but the degree of response varies depending on species and UAV height^10,29^. In some cases, animal disturbance to UAVs is lower than that of traditional survey methods^4^, and other animals demonstrate no behavioral response to UAVs^3,9,13^. For our study, we flew in low wind conditions, had appropriate FAA certificates and daylight exemption waivers, and operated under a New Mexico Department of Game and Fish permit (see Methods for details). Additionally, we adhered to self-imposed guidelines: we had a minimum of two visual observers on our UAV at all times, plus a ground observer that monitored bat behavioral response via thermal imagery. Because (according to our knowledge) this was the first time a UAV had been flown in close proximity to bats, we established a conservative criterion for UAV operation: all flights would be aborted and the project ended if bats collided with the drone and/or exhibited a strong behavioral avoidance response. We defined “strong” as maintaining a 20 m or greater distance away from the drone during flight. Thermal video monitoring revealed bats showed no such strong response to the drone during flight (Fig. 1) and avoided the drone as they would any object during flight, resulting in no collisions during our experiment. We encourage all pilots utilizing UAVs for research to consider and develop conservative standard operating procedures, and carefully research local UAV regulations prior to flight.

## Methods

Data were collected from 24 May to 5 June 2017 at a natural cave structure located on privately owned property in Sierra County, New Mexico, containing a maternal *Tadarida brasiliensis* population in the hundreds of thousands^30^. Each morning between approximately 0300 and 0500, an omnidirectional microphone (Ultramic-250, Dodotronics, Castel Gandolfo, Italy) was affixed to a UAV (DJI SPREADING WINGS S900 Hexacopter Shenzhen, China). The recording system was designed to minimize received noise from the UAV while not compromising flight performance (see^31^ for detailed information on the UAV design and acoustic testing). All methods were carried out in accordance with relevant guidelines and regulations. To fly the UAV during the darkness, we obtained a United States FAA commercial UAV pilot license (issued to LNK) and received a 14 CFR §107.29 Daylight Operation waiver (UAV registration number: FA3ANMN7EX, waiver number: 107W-2017-01361). Field bat recordings were conducted under New Mexico Department of Game and Fish Authorization Number 3651 issued to LNK. All experimental protocols were approved by the Saint Mary’s College Institutional Animal Care and Use Committee.

During all recordings, two observers maintained visual contact with the UAV at all times. The bats and UAV were monitored by a third observer using ground-based thermal imagery. Per our safety protocol, UAV operations could only occur during wind speeds less than 10 km/h, and all UAV activities would cease if bat collision occurred and/or bats exhibited a strong behavioral response, defined as moving a minimum of 20 m away from the UAV.

The UAV was powered on and piloted to a height of five meters directly above the edge of the cave opening, where it hovered to record acoustic data for one minute. After the one minute period had elapsed, the UAV maneuvered to ten meters and recorded for an additional minute. This was repeated until a one minute recording was completed at 5 m, 10 m, 20 m, 30 m, 40 m, and 50 m elevation. The operator then landed the UAV, replaced the battery, and repeated the sampling procedure as described above. Sound files from each recording session were uploaded to *Audacity* version 2.1.3 (Open Source, Audacityteam.org) and calls were identified and extracted via visual inspection of spectrograms.

To ensure each isolated call came from a different bat, we selected calls for further analysis only if they were separated in our recordings by greater than 2 seconds. Based on the only published estimated average flight speed during re-entry of 7.78 m/s^15^, this time interval would ensure other bats were approximately 15.56 m from our UAV, which would result in a reduction in amplitude of 24 dB relative to the true bat call. Additionally, the signal to noise ratio (SNR) of each signal was calculated and calls were selected for final analysis if the SNR was ≥3 dB, which corresponds to double the power of background noise^32^, and further ensures we were not selecting duplicate calls from the same bat.

The final call subsets were analyzed using two custom algorithms written in the MATLAB environment (Mathworks, Natick, MA, USA): one that extracts parameters from the fundamental harmonic of the spectrogram, and another that characterizes and compares the shape of the FM call. First, calls were filtered using a 16 kHz, 8th order high-pass Butterworth filter. For each call, we determined the start and end frequency (defined as the frequency that was −20 dB above and below, respectively, relative to the peak dB of the signal, Fig. 1) and the call duration, which was determined relative to the time values corresponding to the start and stop frequencies. To compare call shapes, we used a second custom algorithm designed to extract and categorize the time-frequency components of echolocation calls via derivative dynamic time warping and agglomerative hierarchical clustering^33^. This method characterizes the call curvature instead of absolute start frequency/end frequency/duration parameters. Because of the differences in our two analysis algorithms (see ref.^33^ for more information), we expect slight differences in the start/end frequency and duration values between Figs 2 and 3. All statistical analyses were completed in SPSS (v23, IBM, Armonk, North Castle, NY, USA). We used Levene’s test for homogeneity of variance for all call parameters across altitudes, and tested the effect of altitude on start frequency, end frequency, and duration using Kruskal-Wallis tests and Mann-Whitney U comparisons.

### Data Availability

Data from this study are available from the Dryad Digital Repository (URL will be available upon article acceptance and/or reviewer request).
